# Development and validation of a novel nomogram integrating visceral-to-subcutaneous fat ratio and neutrophil-percentage-to-albumin ratio for overall survival in locally advanced rectal cancer undergoing neoadjuvant radiotherapy

**DOI:** 10.3389/fonc.2026.1923672

**Published:** 2026-09-03

**Authors:** Xueqing Zhang, Jianjian Qiu, Shiji Wu, Xingte Chen, Junxin Wu

**Affiliations:** Department of Radiation Oncology, Clinical Oncology School of Fujian Medical University, Fujian Cancer Hospital, Fuzhou, China

**Keywords:** locally advanced rectal cancer, neoadjuvant radiotherapy, neutrophil-percentage-to-albumin ratio, nomogram, overall survival, visceral-to-subcutaneous fat ratio

## Abstract

**Background:**

Emerging evidence suggests that neutrophils are preferentially recruited to visceral adipose tissue, connecting adipose distribution to systemic inflammation. Visceral fat accumulation and systemic inflammation-nutrition status are known to affect cancer prognosis, yet no existing prognostic model integrates visceral-to-subcutaneous fat ratio (VSR) and neutrophil-percentage-to-albumin ratio (NPAR). This study aimed to develop and validate a cost-effective nomogram incorporating VSR and NPAR to predict overall survival (OS) for locally advanced rectal cancer (LARC) patients who underwent neoadjuvant radiotherapy (nRT).

**Methods:**

A total of 143 LARC patients receiving nRT followed by total mesorectal excision were retrospectively enrolled. The CT images from radiation CT-Simulator System were used to assess VSR, and routine blood tests were conducted to calculate NPAR and other inflammatory-nutritional markers without extra cost or radiation. LASSO-Cox regression was used to identify independent predictors for OS and a nomogram was constructed and internally validated using 1000 bootstrap resamples. Model performance was assessed through concordance index (C-index), time-dependent receiver operating characteristic (ROC) curves, calibration curves, and decision curve analysis (DCA), and compared with the TNM staging system and pathological complete response (pCR) using discriminant indices.

**Results:**

The median follow-up time was 72.8 months. LASSO-Cox regression identified VSR (HR = 3.755, P = 0.009), NPAR (HR = 3.626, P = 0.003), CA199 (HR = 4.052, P = 0.001), and neural invasion (HR = 2.666, P = 0.047) as independent risk factors for OS. The nomogram showed an original C-index of 0.781, with a bootstrap-corrected value of 0.775. Its AUC values for 3-, 5-, and 8-year OS were 0.816, 0.777, and 0.867, respectively. Calibration curves revealed optimal agreement between predicted and actual observed outcomes, and DCA suggested better clinical net benefits than individual factors. The model demonstrated strong accuracy, discriminative ability, and clinical utility, outperforming TNM staging system and pCR in long-term predictions. Kaplan-Meier analysis demonstrated significantly worse OS in high-risk group (Log-rank P<0.001).

**Conclusion:**

This novel nomogram integrating VSR and NPAR demonstrates favorable long-term prognostic performance for LARC patients following nRT, compared to TNM staging and pCR. Since both markers are obtained from routine radiation simulation CT and blood tests without additional radiation or cost, this cost-effective and accessible model may facilitate risk-based surveillance and personalized therapy in clinical application.

## Introduction

Colorectal cancer (CRC) is the third most common malignancy and the second leading cause of cancer-related mortality worldwide. Rectal cancer accounts for approximately one-third of all CRC cases and is the eighth most prevalent cancer overall (1). Locally advanced rectal cancer (LARC), characterized by transmural rectal invasion (T3 or T4) or regional lymph node metastasis without distant metastasis, remains a major challenge for clinical management. Neoadjuvant radiotherapy (nRT) followed by total mesorectal excision (TME) is the standard treatment for LARC patients. However, the responses to nRT are variable, ranging from pathological complete regression (pCR) to minimal therapeutic effect or even tumor progression (2, 3). While TNM staging reflects cancer invasion, it ignores systemic inflammation and nutrition status. Additionally, pCR indicates short-term response but has limited relevance for long-term survival. Therefore, a prognostic tool integrating risk factors is urgently needed to guide routine clinical practice.

Body mass index (BMI) is the most widely used anthropometric indicator for evaluating obesity and nutritional status. Recent studies have indicated a correlation between obesity and increased incidence and mortality of rectal cancer, whereas the “obesity paradox” still remains controversial (4–6). Compared to BMI, visceral adipose tissue (VAT) is regarded as a more reliable indicator of unhealthy obesity phenotypes (7). VAT influences the function and metabolism of adjacent organs and the tumor microenvironment through the aberrant production of adipokine and pro-inflammatory cytokines (8). Given the distinct biological effects of VAT compared to subcutaneous adipose tissue (SAT), the visceral-to-subcutaneous fat ratio (VSR) is a more effective measure of the adverse metabolic and inflammatory impacts caused by abnormal adipose distribution. Elevated VSR has been demonstrated to correlate with increased all-cause mortality and obesity-associated complications in multiple cancers (9, 10). However, the prognostic value of VSR in LARC patients who underwent nRT remains uncertain.

Systemic inflammation and malnutrition are prevalent among cancer patients and are well-established prognostic factors (11). While indices including systemic immune-inflammation index (SII), systemic inflammation response index (SIRI), and geriatric nutritional risk index (GNRI) have been used for prognosis prediction in rectal cancer (12–14), they may not fully reflect the complex interplay between inflammation and nutrition. The neutrophil-percentage-to-albumin ratio (NPAR), calculated as neutrophil percentage divided by serum albumin levels, simultaneously quantifies systemic inflammation and nutritional depletion (15). Recent studies indicate that NPAR is a cost-effective and promising biomarker in CRC (16, 17), but its prognostic value in LARC patients who underwent nRT has not been fully elucidated.

A recent study demonstrated that neutrophils are more preferentially recruited to visceral fat than subcutaneous fat under sympathetic activation, aiding energy storage in adipocytes (18). Chronic inflammation induced by visceral adiposity further accelerates the progression of malnutrition (19), creating a cycle of visceral adiposity, inflammation, and nutrition issues. This indicates that VSR and NPAR represent complementary aspects of host status, and their combination could enhance prognostic evaluation. Nevertheless, no existing model integrates both for clinical application.

Traditional prognostic models for LARC patients undergoing nRT have two key limitations. They often rely on BMI or sarcopenia, neglecting the prognostic value of visceral/subcutaneous fat imbalance. Moreover, they analyze inflammation and nutrition markers separately, rarely adopting the comprehensive indicator NPAR. No prior nomogram has integrated fat distribution and a composite inflammation-nutrition marker for predicting overall survival (OS). Unlike models based on TNM staging or clinicopathological features, our model integrates abdominal adipose phenotype and systemic host status for a comprehensive prognosis. VSR is obtained from radiation CT-Simulator images, and NPAR from routine blood tests, without extra costs or radiation, making it clinically applicable. Therefore, this study aimed to develop and internally validate a practical, cost-effective nomogram that combines VSR and NPAR to improve the individualized prediction of long-term OS in LARC patients after nRT. This model is expected to support risk-stratified follow-up and tailored treatment strategies.

## Materials and methods

### Study design and study population

This retrospective cohort study encompassed consecutive LARC patients who received nRT followed by TME between January 2017 and May 2021. The inclusion criteria required histologically confirmed adenocarcinoma and staging II (T3-4N0) or III (TanyN+) rectal cancer, according to the 8^th^ Edition of the American Joint Committee on Cancer (AJCC) staging system. The exclusion criteria were: (1) presence of distant metastasis at diagnosis; (2) lack of subsequent surgery following neoadjuvant therapy; (3) concurrent or previous history of other cancers; (4) administration of two-dimensional conventional radiotherapy; and (5) incomplete clinical, radiological, or laboratory data. Finally, a total of 143 eligible patients were included. This research followed the Declaration of Helsinki and was approved by the Ethics Committee of Fujian Cancer Hospital (K2026-305-01). Written informed consent was waived due to the retrospective observation study. All data were extracted from electronic medical records and independently verified by two investigators, including baseline demographics, tumor characteristics, treatment regimens, and clinical outcomes.

### Treatment and follow-up

All patients received individualized nRT determined by experienced radiation oncologists. The radiotherapy regimens were delivered using intensity-modulated radiation therapy (IMRT). The clinical target volume (CTV) included the primary tumor and the anorectal, mesorectal, presacral, and internal iliac lymph nodes. A margin was added for potential movement and setup variability, while organs at risk (pelvic bones, bladder, and small bowel) were carefully spared. Three standardized neoadjuvant treatment protocols were implemented: short-course radiotherapy (SCRT), long-course radiotherapy (LCRT), and total neoadjuvant therapy (TNT). The SCRT protocol administered a total radiation dose of 25 Gy in five fractions. The LCRT protocol consisted of 50 Gy in 25 fractions, with concurrent chemotherapy using capecitabine (825mg/m^2^), administered twice daily, five days per week. The TNT protocol included induction and/or consolidation chemotherapy, such as CAPOX, FOLFOX, and FOLFOXIRI, in combination with neoadjuvant (chemo)radiotherapy before surgery. All patients underwent TME performed by experienced surgeons.

Patients underwent follow-up examinations every three months within the initial two years, every six months for the following three years, and annually thereafter. The last follow-up date was January 15, 2026. OS was defined as the time from diagnosis until death from any cause or the last follow-up.

### CT anthropometric measurements

All CT images were routinely obtained from radiation CT-Simulator System for radiotherapy planning, without extra visits, radiation exposure, or medical expense. Analysis was conducted at the level of the third lumbar vertebra (L3) using SliceOmatic software. VAT, SAT, and skeletal muscle were delineated based on Hounsfield Unit (HU) thresholds of -150 to -50 HU, -190 to -30 HU, and -29 to 150 HU, respectively. The indices were calculated as follows: visceral adipose tissue index (VATI)=visceral adipose area (cm^2^)/height^2^ (m^2^), subcutaneous adipose tissue index (SATI)=subcutaneous adipose area (cm^2^)/height^2^ (m^2^), and skeletal muscle index (SMI)=total muscle area (cm^2^)/height^2^ (m^2^). VSR was calculated as VATI divided by SATI. Sarcopenia was defined by the L3 SMI thresholds: 52.4 cm^2^/m^2^ for males and 38.5 cm^2^/m^2^ for females (20). Central Obesity was defined as waist circumference: ≥102 cm for males and ≥ 88 cm for females (21).

### Calculation of inflammatory and nutritional indicators

Baseline laboratory data were collected at diagnosis to assess patients’ inflammatory and nutritional status. Inflammatory indices were chosen because systemic inflammation during tumor progression affects survival by promoting angiogenesis and suppressing immunity. The systemic immune-inflammation index (SII=N*PL/L) reflects the balance between pro-tumor (neutrophils, platelets) and anti-tumor (lymphocytes) immunity, with higher SII suggesting a tumor-friendly environment. The systemic inflammatory response index (SIRI=N*M/L) assesses the balance between myeloid cells (neutrophils, monocytes) and lymphocytes, linked to tumor-related inflammation. Rationale for choosing nutritional indices: advanced rectal cancer is associated with cachexia and malnutrition, impacting treatment tolerance and survival. The geriatric nutritional risk index (GNRI = 1.489*ALB+41.7*(W/IW)) evaluates the gap between actual and ideal nutritional status using albumin and weight ratios. IW (Ideal weight) = 22*H^2^, if the actual weight exceeds ideal weight, the actual weight/ideal weight=1. The advanced lung cancer inflammation index (ALI=BMI*ALB/neutrophil-to-lymphocyte ratio), uniquely synthesizes nutritional status, reflects the nutrition-inflammation balance. The neutrophil-percentage-to-albumin ratio (NPAR=N(%)/ALB) examines inflammatory cell distribution and nutritional-hepatic reserve, highlighting the relationship between systemic inflammation and nutrition. N, neutrophil; PL, platelet; L, lymphocyte; M, monocyte; ALB, albumin; W, weight; H, height.

### Statistical analysis

Statistical analyses were conducted using R software (Version 4.5.2). A restricted cubic spline (RCS) Cox regression was initially performed to assess the linearity. Given the non-significant test for nonlinearity, receiver operating characteristic (ROC) curve analysis was conducted to assess the predictive value of VSR, SII, SIRI, GNRI, ALI, and NPAR on 3-year OS as a clinically relevant endpoint. The optimal cutoff value was determined by maximizing specificity and sensitivity using Youden’s Index. Due to gender differences in adipose tissue distribution, sex-specific cutoffs for VSR were calculated to prevent cross-gender misclassification and ensure accurate prognostic stratification. Univariate Cox regression analysis was applied for initial feature selection (P<0.1). Least absolute shrinkage and selection operator (LASSO) regression with 10-fold cross-validation was employed to further eliminate variables and mitigate model overfitting. The variables retained after these steps were included in multivariate Cox regression analysis to identify independent prognostic factors (P<0.05). A nomogram was constructed based on the independent predictors and internally validated with 1000 bootstrap resamples. The predictive performance was evaluated using the concordance index (C-index) and time-dependent ROC curves. Calibration curves and the Hosmer–Lemeshow test were employed to evaluate the model’s adequacy in fitting the data, and decision curve analysis (DCA) was developed to assess clinical net benefit. The net reclassification improvement (NRI) and integrated discrimination improvement (IDI) were calculated to quantitatively compare the prognostic performance of our model against traditional TNM staging system and pCR. Finally, to classify participants into low- and high-risk groups, the surv_cutpoint function from the survminer package was utilized to identify the optimal risk score cutoff for the strongest survival outcome association. The Kaplan-Meier method and Log-rank test were utilized to compare survival differences for OS. All analyses were two-sided, with significance at P<0.05.

## Results

### Clinical characteristics

After strict exclusions, a total of 143 patients were enrolled in this retrospective study. **Table 1** displays baseline characteristics. The mean age was 57.23 ± 10.47 years, and 65% of patients were male. Obesity (BMI≥25kg/m^2^) was diagnosed in 35 individuals (24.5%), sarcopenia in 90 (62.9%), and central obesity in 9 (6.3%). Elevated CA199 (>35U/mL) were found in 18 cases (12.6%), and 63 patients (44.1%) had elevated carcinoembryonic antigen (CEA, >5ng/mL). Approximately 44.1% of patients had low-lying tumors (within 5 cm from the anal verge), 74 cases (51.7%) showed mesorectal fascia (MRF) involvement, and 89 patients (62.2%) had extramural vascular invasion (EMVI). The cT stage distribution was cT2 (4 cases), cT3 (97 cases), and cT4 (42 cases), with 131 cases (91.6%) having lymph node metastasis. After nRT, 90 patients (62.9%) had pathological (yp) T3-T4 tumors, and 87 (60.8%) were ypN0. Lymphovascular invasion (LVI) was seen in 47 patients (32.9%), and neural invasion (NI) in 23 patients (16.1%). In neoadjuvant therapy, 40 patients (28.0%) received LCRT, 28 patients (19.6%) underwent SCRT, and 75 patients (52.4%) had TNT, with no significant baseline differences among these subgroups (**Supplementary Table 1**). A total of 25 patients (17.5%) achieved pCR, with 7 cases in the LCRT group and 18 in TNT group. Adjuvant chemotherapy was administered to 118 patients (82.5%). The RCS Cox regression showed no nonlinearity between VSR and OS (overall population P for nonlinearity = 0.102; males P for nonlinearity = 0.285; females P for nonlinearity = 0.594). The cutoff value for VSR was 1.413 in males and 0.759 in females. Optimal cutoff values for SII, SIRI, GNRI, ALI, and NPAR are 789.68, 0.94, 114.34, 939.91, and 17.32, respectively. The median follow-up was 72.8 months.

**Table 1:** Characteristics of the participants.

Characteristics	Classifications	No. of patients
Age (mean ± SD)		57.23 ± 10.47
Gender (%)	Female	50 (35.0)
	Male	93 (65.0)
BMI (mean ± SD)		22.63 ± 3.14
Obesity (%)	No	108 (75.5)
	Yes	35 (24.5)
SMI (mean ± SD)		44.81 ± 7.74
Sarcopenia (%)	No	53 (37.1)
	Yes	90 (62.9)
Waist (mean ± SD)		79.72 ± 8.80
Central Obesity (%)	No	134 (93.7)
	Yes	9 (6.3)
SATI (mean ± SD)		37.12 ± 24.59
VATI (mean ± SD)		26.24 ± 20.41
VSR (%)	Low	121 (84.6)
	High	22 (15.4)
SII (%)	>789.68	44 (30.8)
	≤789.68	99 (69.2)
SIRI (%)	>0.94	65 (45.5)
	≤0.94	78 (54.5)
GNRI (%)	>114.34	10 (7.0)
	≤114.34	133 (93.0)
ALI (%)	>939.91	8 (5.6)
	≤939.91	135 (94.4)
NPAR (%)	≤17.32	104 (72.7)
	>17.32	39 (27.3)
CA199 (U/mL, %)	≤35	125 (87.4)
	>35	18 (12.6)
CEA (ng/mL, %)	≤5	80 (55.9)
	>5	63 (44.1)
Distance to anal margin (%)	High	4 (2.8)
	Middle	76 (53.1)
	Low	63 (44.1)
Mesorectal fascia (%)	No	69 (48.3)
	Yes	74 (51.7)
Extramural vascular invasion (%)	No	54 (37.8)
	Yes	89 (62.2)
TNM Stage (%)	II	20 (14.0)
	III	123 (86.0)
cT stage (%)	T2	4 (2.8)
	T3	97 (67.8)
	T4	42 (29.4)
cN stage (%)	N0	12 (8.4)
	N1-2	131 (91.6)
Neoadjuvant therapy (%)	LCRT	40 (28.0)
	SCRT	28 (19.6)
	TNT	75 (52.4)
Neoadjuvant chemotherapy (%)	No	28 (19.6)
	Yes	115 (80.4)
Pathologic differentiation (%)	Highly and moderately	109 (87.2)
	Poorly	16 (12.8)
ypT stage (%)	T0	25 (17.5)
	T1	2 (1.4)
	T2	26 (18.2)
	T3	81 (56.6)
	T4	9 (6.3)
ypN stage (%)	N0	87 (60.8)
	N1	40 (28.0)
	N2	16 (11.2)
Lymphovascular invasion (%)	No	96 (67.1)
	Yes	47 (32.9)
Neural invasion (%)	No	120 (83.9)
	Yes	23 (16.1)
pCR (%)	No	118 (82.5)
	Yes	25 (17.5)
Adjuvant chemotherapy (%)	No	25 (17.5)
	Yes	118 (82.5)

BMI, body mass index; SMI, skeletal muscle index; SATI, subcutaneous adipose tissue index; VATI, visceral adipose tissue index; VSR, visceral-to-subcutaneous fat ratio; SII, systemic immune-inflammation index; SIRI, systemic inflammatory response index; GNRI, geriatric nutritional risk index; ALI, advanced lung cancer inflammation index; NPAR, neutrophil-percentage-to-albumin ratio; CEA, carcinoembryonic antigen; LCRT, long-course radiotherapy; SCRT, short-course radiotherapy; TNT, total neoadjuvant therapy; pCR, pathological complete response.

### Predictive indicators selection

**Figure 1:**
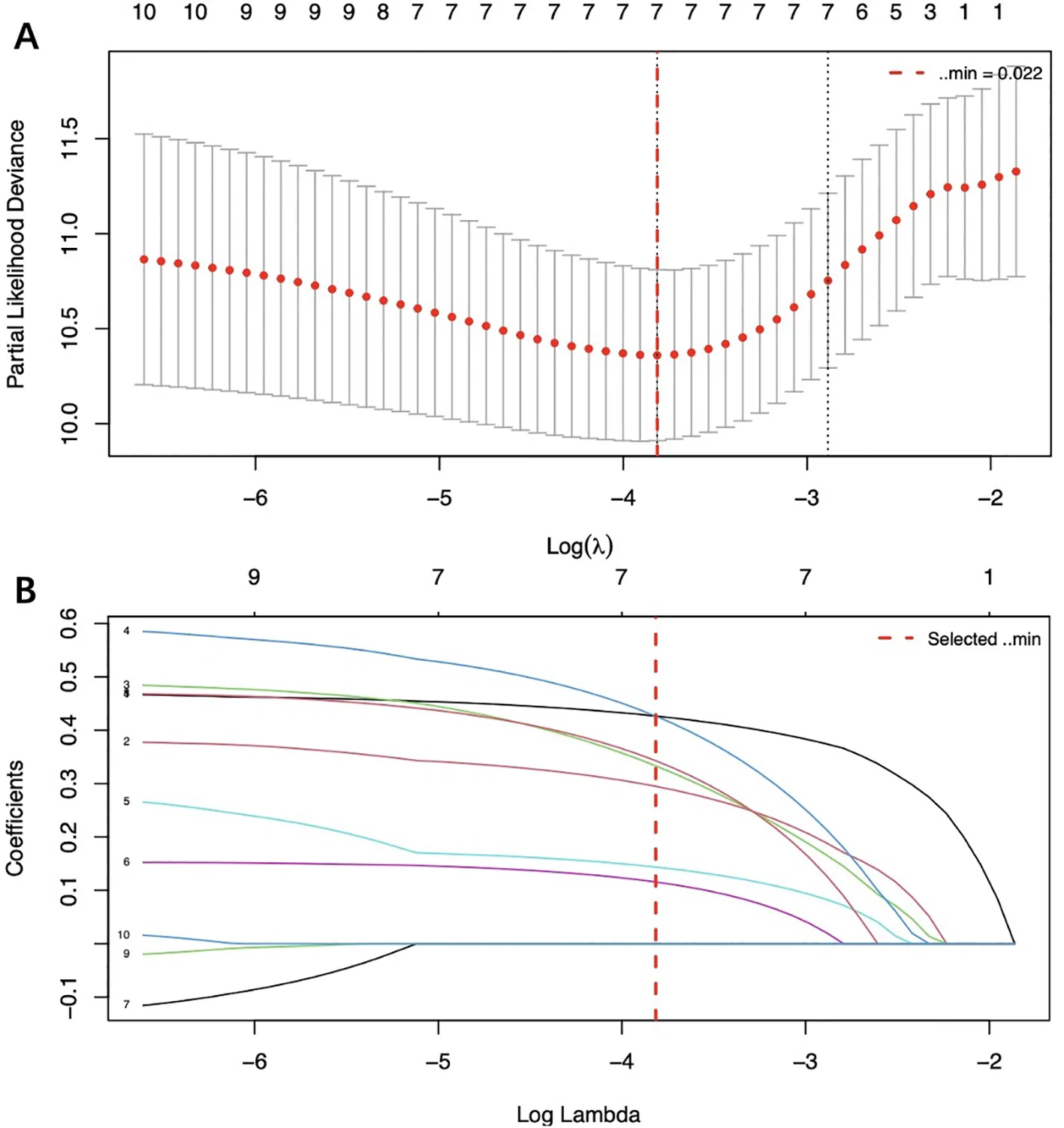
Feature selection utilizing LASSO regression. **(A)** Determination of the optimal penalty parameter (λ) using 10-fold cross-validation, employing the minimum criterion. The vertical dashed line represents the optimal λ value (λ.min). **(B)** LASSO coefficient profiles illustrating the seven optimal predictors identified at the selected log(λ) value.

Univariate Cox regression analysis identified factors associated with OS, including central obesity, VSR, SII, SIRI, NPAR, CA199, ypT stage, ypN stage, LVI, NI, and pCR (P<0.1). To address potential multicollinearity, variables with P<0.1 from univariate analysis were subjected to LASSO regression with 10-fold cross-validation. Although SII, SIRI, LVI, and pCR were identified in univariate analysis, they were excluded from the final model as LASSO reduced their coefficients to zero. **Figure 1** shows that central obesity, VSR, NPAR, CA199, ypT, ypN, and NI were key predictors with non-zero coefficients. These were incorporated into the multivariate Cox regression model for prognostic evaluation. As presented in **Table 2**, multivariate Cox results demonstrated that VSR [(Hazard Ratio), HR], 3.755; 95% Confidence interval (CI), 1.393-10.122; P = 0.009], NPAR (HR, 3.626; 95%CI, 1.537-8.554; P = 0.003), CA199 (HR, 4.052; 95%CI, 1.758-9.341; P = 0.001), and NI (HR, 2.666; 95%CI, 1.012-7.023; P = 0.047) constituted independent prognostic indicators for OS.

**Table 2:** Univariate and multivariate Cox regression analysis of prognostic factors for overall survival.

Characteristics	Univariate			Multivariate		
	HR	95% CI	P	HR	95% CI	P
Central Obesity						
Yes/No	3.312	1.140-9.621	0.028	1.903	0.555-6.527	0.306
VSR						
High/Low	2.437	1.022-5.809	0.044	3.755	1.393-10.122	0.009
SII						
>789.68/≤789.68	1.958	0.905-4.236	0.088			
SIRI						
>0.94/≤0.94	2.045	0.928-4.510	0.076			
NPAR						
>17.32/≤17.32	2.808	1.301-6.060	0.009	3.626	1.537-8.554	0.003
CA199						
>35/≤35	5.625	2.542-12.446	<0.001	4.052	1.758-9.341	0.001
ypT stage						
T3-4/T0-2	5.166	1.550-17.211	0.008	2.842	0.746-10.834	0.126
ypN stage						
N1-2/N0	2.818	1.278-6.218	0.010	1.445	0.602-3.466	0.410
Lymphovascular invasion						
Yes/No	2.298	1.065-4.961	0.034			
Neural invasion						
Yes/No	3.951	1.752-8.909	<0.001	2.666	1.012-7.023	0.047
pCR						
Yes/No	0.180	0.024-1.329	0.093			

HR, Hazard ratio; CI, Confidence interval; VSR, visceral-to-subcutaneous fat ratio; NPAR, neutrophil-percentage-to-albumin ratio; SII, systemic immune-inflammation index; SIRI, systemic inflammatory response index; pCR, pathological complete response.

### Construction and validation of nomogram

**Figure 2:**
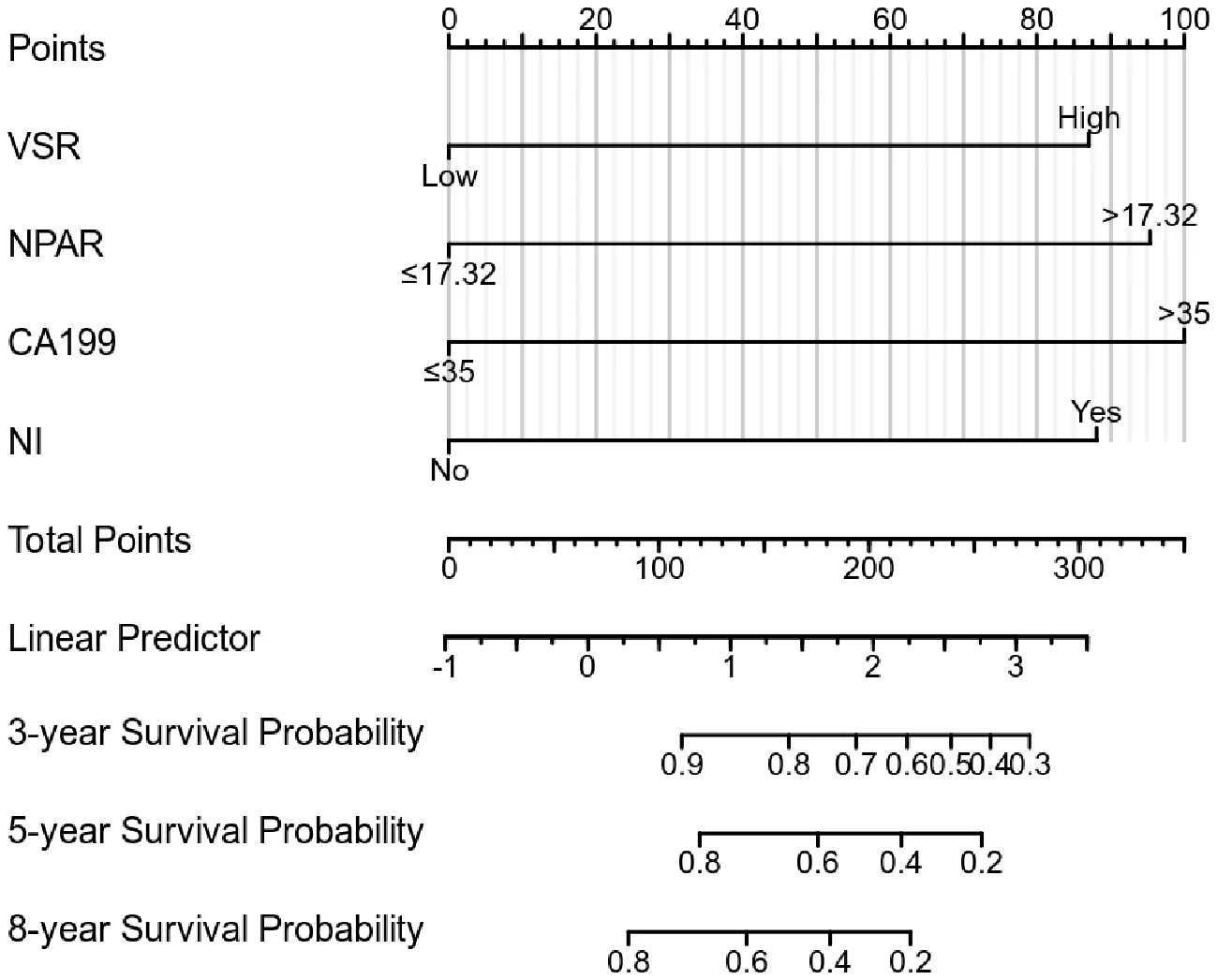
Prognostic nomogram for predicting 3-, 5-, and 8-year overall survival. VSR, visceral-to-subcutaneous fat ratio; NPAR, neutrophil-percentage-to-albumin ratio; NI, neural invasion.

**Figure 3:**
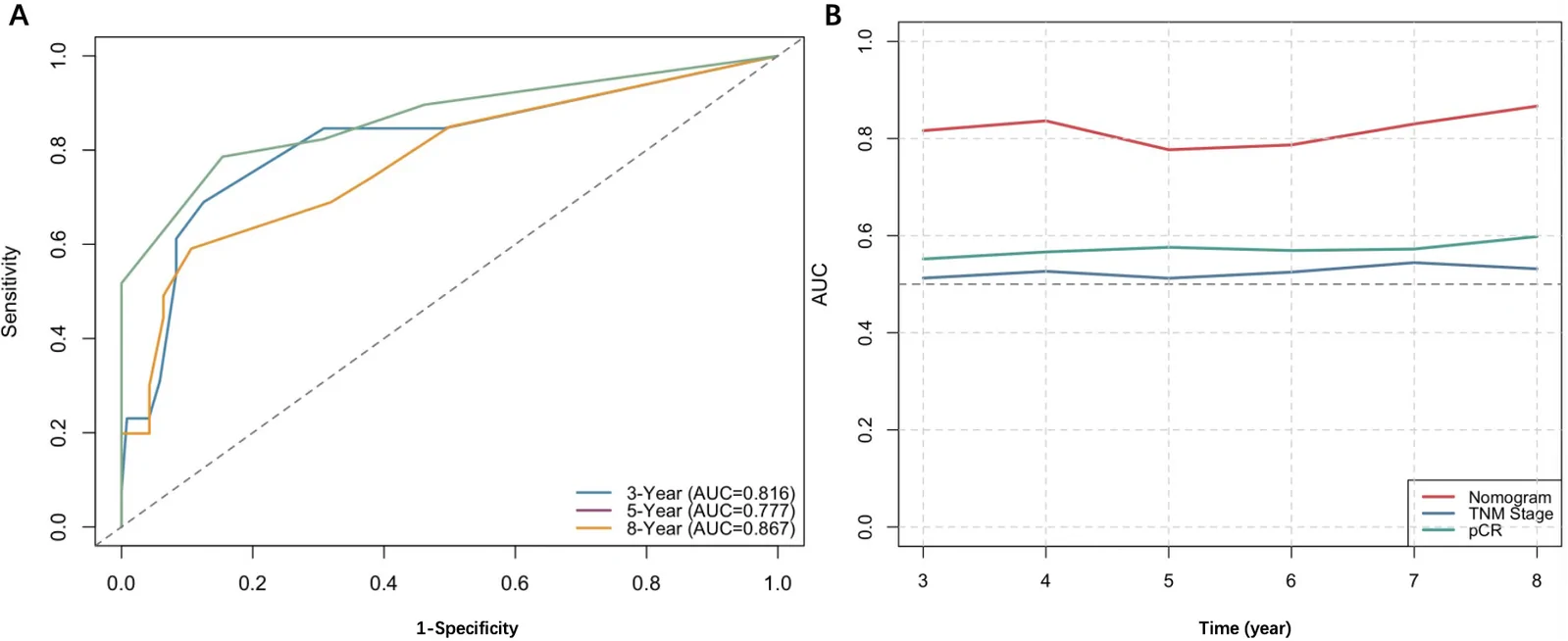
**(A)** Time-dependent ROC curves assessing the predictive performance of the nomogram for long-term survival. **(B)** Time-dependent AUC of the model compared to the conventional TNM staging system and pathological complete regression (pCR).

**Figure 4:**
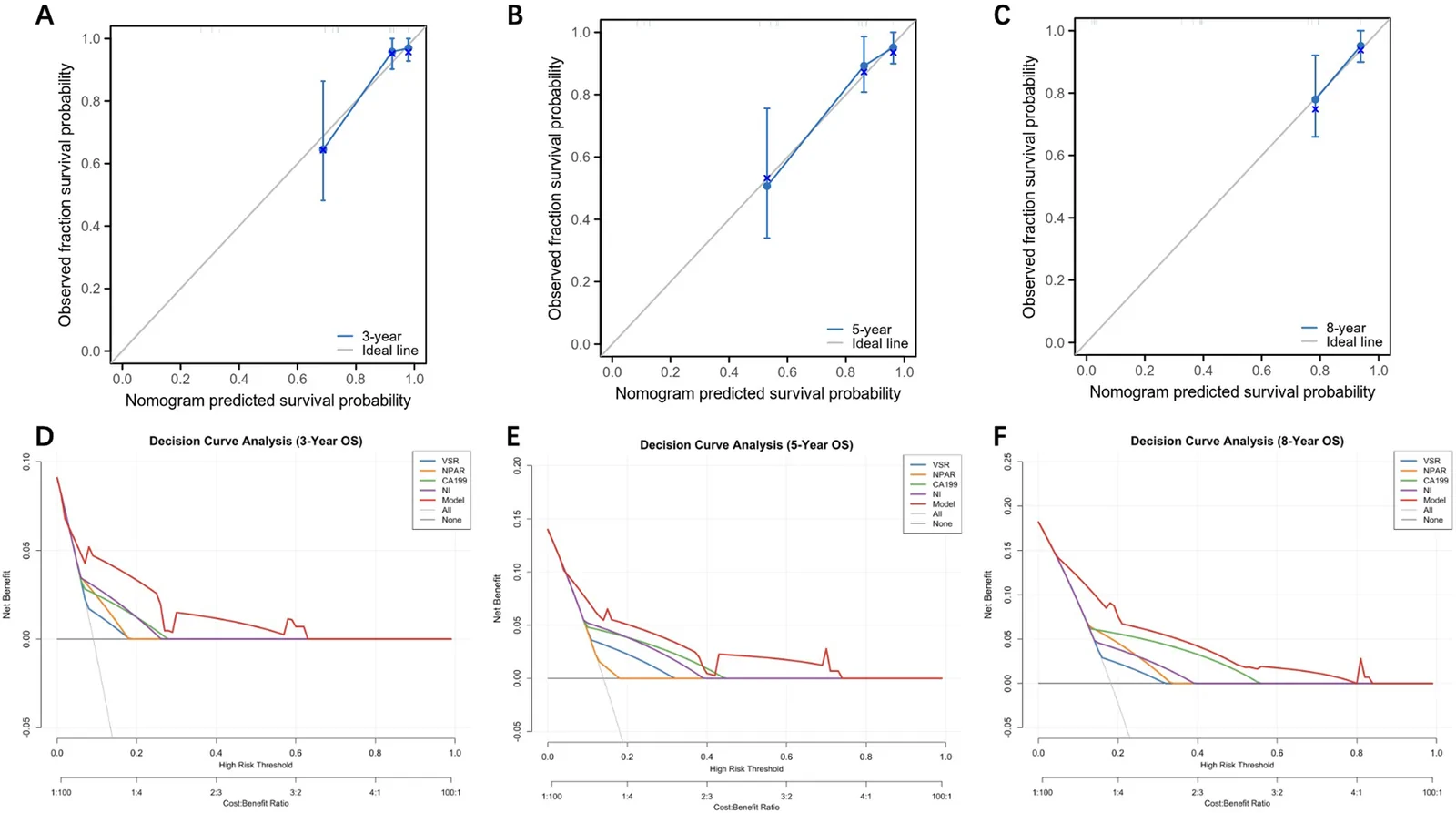
Calibration and clinical utility evaluation of the nomogram model. Calibration curves for 3-, 5-, and 8-year overall survival **(A–C)**. Decision curve analysis for 3-, 5-, and 8-year overall survival **(D–F)**. These curves demonstrated the range of threshold probabilities where the net benefit was positive, indicating clinically relevance.

A nomogram incorporating VSR, NPAR, CA199, and NI was developed to predict OS of LARC patients who underwent nRT, exhibiting strong discriminatory power (**Figure 2**). Bootstrap resampling with 1000 iterations was performed for internal validation. The original C-index of the nomogram was 0.781, and the bootstrap-corrected C-index was 0.775. Time-dependent ROC curve analysis indicated that the AUC values were 0.816, 0.777, and 0.867 for 3-, 5- and 8-year OS, respectively (**Figure 3A**). Calibration curves demonstrated excellent concordance between predicted and observed survival probabilities indicating good model calibration (**Figures 4A–C**). The Hosmer–Lemeshow test indicated that the predictive model was well calibrated for 3-year (χ2 = 4.479, P = 0.214), 5-year (χ2 = 3.511, P = 0.319), and 8-year (χ2 = 3.259, P = 0.353). DCA further highlighted the nomogram’s clinical utility, showing a clear net benefit in predicting 3-, 5- and 8-year OS (**Figures 4D–F**).

Time-dependent ROC curve analysis indicated that the nomogram provided a marked improvement over the conventional TNM staging system and pCR in predicting long-term survival, especially with extended follow-up (**Figure 3B**). At 8-year OS, the nomogram’s AUC was 0.867, outperforming TNM staging (AUC = 0.532) and pCR (AUC = 0.598). Quantitative evaluation using the C-index, NRI, and IDI further confirmed its excellent prognostic accuracy (**Table 3**), with the nomogram achieving a C-index of 0.781, compared to 0.516 for clinical TNM staging and 0.571 for pCR. At 3 years, the nomogram showed improved IDI over TNM staging (IDI = 0.216, 95%CI=0.071-0.474, P = 0.007) and pCR (IDI = 0.208, 95%CI=0.060-0.456, P = 0.007), although NRI was not significant. At 5 and 8 years, both IDI and NRI demonstrated favorable significant improvements. For TNM staging, the 5-year NRI was 0.455 (95%CI=0.202-0.708) and IDI was 0.252 (95%CI=0.124-0.462, P<0.001), while 8-year NRI was 0.458 (95%CI=0.224-0.672) and IDI was 0.307 (95%CI=0.151-0.465, P<0.001). For pCR, the 5-year NRI was 0.455 (95%CI=0.202-0.708) and IDI was 0.234 (95%CI=0.116-0.439, P<0.001), and the 8-year NRI was 0.458 (95%CI=0.224-0.672) and IDI was 0.299 (95%CI=0.139-0.466, P<0.001).

**Table 3:** Comparison of discriminatory ability of nomogram with TNM stage and pCR for LARC patients.

Comparison	Time point	NRI			IDI	
		Difference	NRI+	NRI-	Difference	P
Nomogram vs TNM stage	3-year	0.190 (-0.030-0.407)	0.235	-0.045	0.216 (0.071-0.474)	0.007
	5-year	0.455 (0.202-0.708)	0.534	-0.078	0.252 (0.124-0.462)	<0.001
	8-year	0.458 (0.224-0.672)	0.505	-0.048	0.307 (0.151-0.465)	<0.001
Nomogram vs pCR	3-year	0.190 (-0.030-0.407)	0.235	-0.045	0.208 (0.060-0.456)	0.007
	5-year	0.455 (0.202-0.708)	0.534	-0.079	0.234 (0.116-0.439)	<0.001
	8-year	0.458 (0.224-0.672)	0.505	-0.048	0.299 (0.139-0.466)	<0.001

pCR, pathological complete response; LARC, locally advanced rectal cancer; NRI, net reclassification improvement; IDI, integrated discrimination improvement.

### Risk stratification and survival analysis

**Figure 5:**
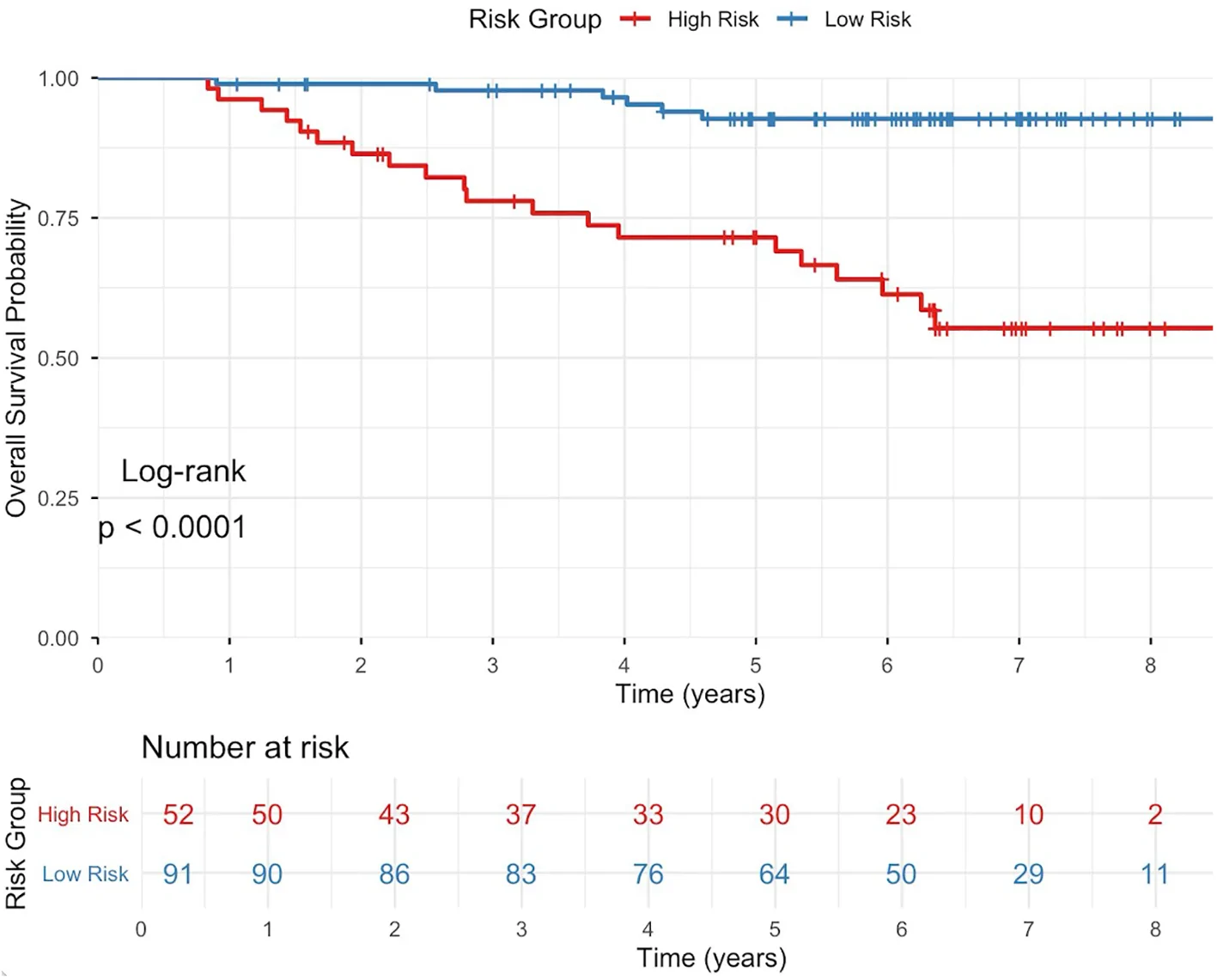
Kaplan-Meier overall survival curves stratified by the risk score derived from the nomogram model.

Using the optimal risk score cutoff value of 1.297, identified as corresponding to the most significant association with survival based on maximally selected rank statistics, all patients were divided into low-risk and high-risk subgroups. Kaplan-Meier survival analysis demonstrated that the high-risk group had significantly poorer OS than the low-risk group (Log-rank P<0.0001, **Figure 5**). Further subgroup analyses exhibited substantial survival differences between high- and low-risk cohorts in various subgroups, such as age, gender, sarcopenia, and clinical TNM staging (All Log-rank P<0.05, P for interaction>0.05, **Supplementary Figure 1**). These findings indicated that the nomogram model, incorporating visceral obesity and inflammatory-nutritional status, demonstrated strong risk stratification capabilities for LARC patients after nRT.

## Discussion

To our knowledge, no prognostic nomogram combines visceral adipose accumulation (VSR) and systematic inflammation-nutrition status (NPAR) for LARC patients undergoing nRT. This study addresses this gap by using CT-derived body composition and routine laboratory parameters, enhancing clinical application without extra cost or radiation exposure compared to radiomics or expensive biomarkers. Previous studies on rectal cancer have focused on individual indicators. For instance, a high VSR was reported to be strongly correlated with elevated postoperative complications (22). NPAR was found as an independent predictor of OS in CRC (16). Our study identified VSR, NPAR, CA199, and NI as significant predictors of OS and developed a practical dynamic nomogram to stratify high-risk populations and improve individualized follow-up strategies.

Obesity is a well-established risk factor for various cancers including rectal cancer. VAT is closely associated with the early recurrence of cancer, and its predictive capability is assessed to outperform SAT (23). Recent study has explored the VSR as a promising prognostic biomarker (24), given that VAT exhibits greater metabolic activity than SAT (25). Increasing evidence suggests that VSR is more accurate than BMI in reflecting CRC pathogenesis (26). Body composition is reported to link to tumor microenvironment characteristics, including stromal composition, epithelial-mesenchymal transition (EMT)-related pathways, and immune cell infiltration (27). As an active secretory organ, adipose tissue produces both proinflammatory and anti-inflammatory cytokines and adipokines. SAT mainly exhibits anti-inflammatory properties, in contrast to the proinflammatory nature of VAT, which has been implicated in various cancers. This biological difference elucidates why BMI cannot serve as a reliable prognostic marker, as it fails to distinguish fat distribution and muscle mass (28). To the best of our knowledge, this is the first study to specifically assess VSR as an indicator of adipose tissue distribution, particularly focusing on the differential levels of visceral and subcutaneous fat in LARC patients after nRT, and explore its impact on long-term OS. Our analysis showed that VSR was an independent and significant prognostic factor (HR = 3.755, 95%CI=1.393–10.122, P = 0.009). VSR showed better predictive performance than BMI. By comparison, central obesity had no significant prognostic value after multivariate analysis (HR = 1.903, 95% CI = 0.555-6.527, P = 0.306). Consistent with previous research, VSR was a stronger prognostic indicator than total body fat or visceral fat alone (29). BMI fails to distinguish fat mass from lean mass, while central obesity only provides crude morphological evaluation. In contrast, VSR reflects visceral fat distribution, which is associated with tumor-related inflammation. This makes VSR a more precise risk stratification tool for LARC patients. Consequently, targeted nutritional interventions to reduce VAT and lower VSR may effectively improve the long-term prognosis of LARC patients after nRT.

VAT plays a key role in immune modulation by secreting adipokines (e.g., leptin, adiponectin, and resistin) and inflammatory cytokines (e.g., TNF-α, IL-1, IL-6, and IL-12), and proinflammatory neutrophils is recruited (8). Recent research highlights neutrophils are crucial in energy storage regulation in sympathetically activated adipocytes, with a more significant increase in VAT compared to SAT (18). This preferential recruitment of neutrophils may explain the association between elevated visceral adiposity (VSR), and increased NPAR, as neutrophil percentage increases and systemic inflammation escalates. Chronic inflammation activated by VAT may also contribute to malnutrition and influence albumin metabolism (19), further raising NPAR. VSR indicates abnormal adipose distribution, while NPAR quantifies inflammation and nutritional depletion, both potentially forming a visceral adiposity-inflammation-nutrition axis affecting outcomes in LARC patients. Elevated VSR and NPAR perpetuate inflammation and nutritional depletion. While individual biomarkers are limited, combining VSR and NPAR offers a comprehensive view of visceral fat’s effects and systemic inflammatory-nutritional status. This study is the first to construct a prognostic model by combining adipose distribution and systemic inflammation-nutrition status to assess their joint impact on long-term LARC survival.

As a composite index integrating both inflammatory and nutritional status, NPAR has garnered increasing attention due to its simplicity and cost-effectiveness. Prior research has demonstrated that NPAR possesses predictive value for cancer incidence, postoperative complications, and survival in several cancers (30–33). High neutrophil levels are indicative of chronic tumor-promoting inflammation, which facilitates tumor angiogenesis, invasion, and an immunosuppressive environment (34). In contrast to absolute neutrophil counts, neutrophil percentage more stably reflects inflammatory status by minimizing variability in total leukocyte counts. Additionally, hypoalbuminemia indicates malnutrition and immune dysfunction, which increases complications risk (16). By integrating these components, NPAR provides a more comprehensive assessment of host status. Compared to the neutrophil to albumin ratio (NAR), which is calculated by dividing neutrophil counts by albumin, NPAR has attracted greater academic interest due to its use of neutrophil percentage for normalization, thereby offering better stability. Previous studies have demonstrated that NAR is an independent predictor of pCR in rectal cancer (odds ratio=0.76, 95% CI 0.60-0.97) (35). However, there remains a significant research gap, as studies focusing on NPAR in LARC patients undergoing nRT are notably limited. Our results revealed that patients with elevated NPAR had significantly poorer OS (HR, 3.626; 95%CI, 1.537–8.554; P = 0.003). In addition, compared with single inflammatory markers (SII and SIRI) or nutritional markers (GNRI and ALI), NPAR exhibited excellent prognostic efficacy in our cohort.

Furthermore, CA199 (HR, 4.052; 95%CI, 1.758–9.341; P = 0.001) and NI (HR, 2.666; 95%CI, 1.012–7.023; P = 0.047) were also identified as independent predictors of OS. CA199 is an established serum tumor biomarker in CRC, indicative of tumor burden and aggressiveness. And NI has been correlated with local recurrence and decreased survival. The incorporation of these factors into the nomogram further enhances its prognostic precision.

Recent nomograms for LARC patients receiving nRT primarily use traditional clinicopathological features, such as TNM staging, LVI and NI, with some incorporating single inflammatory markers or body composition parameters (14, 36, 37). However, none have combined visceral fat accumulation (VSR) and systemic inflammatory-nutritional status (NPAR). Our nomogram, with a C-index of 0.781 and AUCs of 0.816 and 0.777 for 3- and 5-year OS, outperformed the prior nomogram using TNM staging and CEA (C-index = 0.747, AUC = 0.766 and 0.736) (38). Another nomogram using body composition metrics and blood biomarkers had a C-index of 0.763 and AUC of 0.763 for predicting pCR, but did not address long-term OS (39). Firstly, the novel model avoids the need for extra imaging or costly genetic tests, lowering clinical application barriers compared to radiomics or molecular models. Secondly, this nomogram outperforms traditional TNM staging and pCR in prognostic accuracy, helping clinicians identify high-risk patients for personalized management. TNM staging and pCR focus on tumor burden and immediate treatment response, offering short-term predictive value but missing long-term metabolic-inflammatory features. The categorical NRI is influenced by limited early death events within 3 years, whereas continuous IDI remains stable. At 5- and 8-year survival endpoints, with more death events, both NRI and IDI showed significant prognostic superiority over TNM staging and pCR, highlighting the long-term predictive advantage of the novel nomogram. For high-risk patients stratified by the nomogram, intensified postoperative surveillance could be considered, including imaging follow-ups and early interventions like nutritional support and lifestyle changes. Low-risk patients might benefit from reduced surveillance to decrease medical burden. These strategies need further validation in prospective clinical trials.

## Limitation

Several limitations should be acknowledged. Firstly, it was a single-center retrospective study, which may introduce selection bias. Due to excluding patients lacking suitable CT images or feasible segmentation, the cohort size was moderate. Secondly, although variable selection and model stability were enhanced using methods such as LASSO regression, Cox regression, and internal validation, external validation based on independent multicenter cohorts is recommended to verify the nomogram’s generalizability and cutoff values of VSR and NPAR. Thirdly, the retrospective nature of the study limited the analysis to baseline VSR and NPAR, and dynamic changes during treatment were not explored. Therefore, further large-scale prospective studies are needed to address the limitations and confirm the clinical relevance.

## Conclusion

In conclusion, VSR and NPAR are novel, economical, and independent prognostic biomarkers for LARC patients who treated with nRT. The developed nomogram, which incorporates these two readily available metrics together with CA199 and NI, demonstrated a satisfactory predictive capability for 3-, 5-, and 8-year OS. This model has good clinical practicability and may aid in individualized risk stratification and long-term surveillance strategies for LARC patients.

## Data Availability

De-identified human subject data supporting the conclusions of this article will be made available by the authors upon reasonable request.
