# Supplementary material for: Development and validation of a novel nomogram integrating visceral-to-subcutaneous fat ratio and neutrophil-percentage-to-albumin ratio for overall survival in locally advanced rectal cancer undergoing neoadjuvant radiotherapy
Source: Front Oncol. 2026 Sep 3;16:1923672. doi: 10.3389/fonc.2026.1923672 (PMC13581682; doi:10.3389/fonc.2026.1923672)
Supplement: Supplementary file 1 [file DataSheet1.docx]

Supplementary Figure S1. Kaplan-Meier survival curves for overall survival stratified by (A) age, (B) gender, (C) sarcopenia, and (D) clinical TNM stage.
